# Distance-Based Detection of Cough, Wheeze, and Breath Sounds on Wearable Devices

**DOI:** 10.3390/s22062167

**Published:** 2022-03-10

**Authors:** Bing Xue, Wen Shi, Sanjay H. Chotirmall, Vivian Ci Ai Koh, Yi Yang Ang, Rex Xiao Tan, Wee Ser

**Affiliations:** 1 Department of Electrical and Systems Engineering, Washington University in St. Louis, St. Louis, MO 63130, USA; xuebing@wustl.edu; 2 Harvard Medical School, Harvard University, Cambridge, MA 02115, USA; wshi3@mgh.harvard.edu; 3 Lee Kong Chian School of Medicine, Nanyang Technological University, Singapore 637551, Singapore; schotirmall@ntu.edu.sg; 4 Aevice Health Pte. Ltd., Singapore 637551, Singapore; vivian@aevice.com (V.C.A.K.); yiyang@aevice.com (Y.Y.A.); rex@aevice.com (R.X.T.)

**Keywords:** acoustic signal processing, distance classification, feature selection algorithm, health monitoring, wearable devices

## Abstract

Smart wearable sensors are essential for continuous health-monitoring applications and detection accuracy of symptoms and energy efficiency of processing algorithms are key challenges for such devices. While several machine-learning-based algorithms for the detection of abnormal breath sounds are reported in literature, they are either too computationally expensive to implement into a wearable device or inaccurate in multi-class detection. In this paper, a kernel-like minimum distance classifier (K-MDC) for acoustic signal processing in wearable devices was proposed. The proposed algorithm was tested with data acquired from open-source databases, participants, and hospitals. It was observed that the proposed K-MDC classifier achieves accurate detection in up to 91.23% of cases, and it reaches various detection accuracies with a fewer number of features compared with other classifiers. The proposed algorithm’s low computational complexity and classification effectiveness translate to great potential for implementation in health-monitoring wearable devices.

## 1. Introduction

Acoustic signal processing is a growing area of focus in healthcare and biomedicine. The characteristics of acoustic signals, especially that of abnormal breath sounds, provide clinicians with valuable information on respiratory diseases [1]. Traditionally, clinicians use stethoscopes as an indispensable tool for healthcare delivery for their reliability and efficiency in aiding the investigation of bodily sounds. However, the interpretation of breath sounds heard through a stethoscope is user dependent, and its single examination mode of measurement disallows continuous monitoring of daily symptoms and fluctuations [2]. With the progression of sensing technology development, wearable devices are now available as promising solutions. They facilitate automatic and continuous acoustic analysis and assist clinicians in evaluating the effectiveness of a prescribed intervention [3].

Amongst the various breath sounds, wheeze is of particular interest as its presence and duration are a significant reference for clinicians to diagnose and monitor various pulmonary pathologies, including chronic obstructive pulmonary disease (COPD), bronchiolitis, and asthma [4]. In addition to wheezing, cough is another common respiratory sound and significant symptom of interest. Its pattern and changes over time can indicate disease evolution in more than 100 different diseases, and its resolution reflects the effectiveness of therapy [5].

The biological features of cough, wheeze, and breath sounds can be characterized by both the spectral and temporal analysis [6,7]. For example, the wheezing sounds during respiration occur when the airway is obstructed or narrowed; hence, the fundamental frequency of the respiratory sound was of a higher frequency when compared with a normal breath. Furthermore, wheeze is often perceived as musical tone where the harmonics can be observed in the spectral domain. Coughs, although having similar properties as wheezes, such as having a higher fundamental frequency and harmonics, are explosive in nature, whereas wheezes are continuous and more commonly found in the expiration phase rather than the entire respiratory cycle. Given these biological characteristics of wheeze and cough sounds, various classification algorithms were developed to differentiate wheeze and cough signals using their spectral and temporal features. Applying hidden Markov models on the extracted cepstral features from a database of 2155 cough events, Matos et al. [8] achieved 82% detection accuracy in the binary classification of cough and non-cough sound samples. Aydore et al. [9] investigated 246 wheeze and non-wheeze epochs using Fisher’s ratio and Neyman Pearson hypothesis and achieved 93.5% sensitivity. Jain et al. [10] extracted spectral features from 19 wheeze samples and 21 other lung sounds and applied a multi-stage fixed threshold classifier for 85% overall accuracy. Lin et al. [11] developed a bilateral filter-based edge-processing technique for 90 wheeze samples and detected 87 out of them. Time-series deep learning models were also examined in previous studies. For example, Justice Amoh [12] used the convolutional neural network and recurrent neural network for cough detection among 14 subjects and achieved 82.5% and 79.9% overall accuracy, respectively. Arati Gurung [13] reviewed 12 recent studies of automatic abnormal lung sound detection, and the average accuracy was 85%. However, all the above studies focused on the detection of a single symptom; the robustness of such methods is not addressed if applied to multiple concurrent symptoms, a feature of ‘real-world’ clinical practice. To the best of the authors’ knowledge, the only study that explored the overall detection of multiple concurrent symptoms including cough and wheeze was performed by Himanshu S. Markandeya [14]. This system employed a two-stage classification structure and achieved 90% accuracy from 74 recordings. It identified discrete wavelet transform (DWT) coefficients for symptoms and applied a specific processing method for each identified DWT coefficient to determine symptoms. This classification algorithm, however, assumes that the DWT coefficient of wheeze sounds are resolved as $D_{5}$, but another study [15] illustrated that wheeze data exhibits useful information throughout $D_{6}\;$to $D_{2}$. Therefore, using exclusive processing methods for specific DWT coefficients would be unsuitable when the actual symptom is not from its expected category.

Health monitoring wearable devices are designed to simultaneously detect multiple respiratory sounds (and perhaps symptoms) to provide comprehensive health and medical insights to the wearer and healthcare providers. However, previous studies mainly focused on a binary classification problem in cough or wheeze detection. Extending them to multi-class classification requires different model signs and higher model complexity to attain satisfactory detection performance. Moreover, none of these existing models consider the unique patterns (for example, distribution of each class in each feature) of various respiratory sounds. Researchers have long been calling for simple yet effective methods to diagnose and monitor patients on wearable devices [16].

This study addresses the challenges of existing cough or wheeze detection studies, attempting to find an optimal acoustic signal processing method for implementation in a wearable device. The proposed method includes a kernel-like input mapping strategy by first transforming the original sound signals in time series to a higher dimensional feature space that characterizes the temporal and spectral patterns of respiratory sounds, followed by a tailored dimension reduction for the algorithm to achieve robustness and generalizability by distilling the prediction information from only a few features. Since data processing is one of the main consumers of battery power in wearable devices [17], the superior prediction performance and minimized feature extraction in the proposed method would make such devices more suitable in clinical applications.

This paper is organized as follows. Section 2 describes the collected dataset, the proposed data-processing methods, and its unique features. It also includes a short discussion on its implementation on an embedded system. In Section 3, the results from the proposed data processing methods are presented and analyzed. Finally, the findings are discussed in Section 4.

## 2. Materials and Methods

### 2.1. Data

For the generalizability of the study, raw data were collected from a variety of sources, as illustrated in Table 1. These sound signals were recorded using different devices with a large sampling frequency and noise-signal ratio variation. Hospital patients’ data were collected either in the outpatient clinic or in-patient ward of a hospital in Singapore, with informed consent obtained from all participants. Respiratory sounds were recorded over the right side of the chest wall using an electret microphone with a sampling rate of 8 kHz and a dedicated cavity for air-coupling purposes. Other data types were included in the final analysis. The open-source datasets were selected from readily available sound libraries or published works [18]. Participant subjects were volunteers who contributed breathing or coughing sounds by recording with their own devices. In this project, 604 segments of acoustic data were acquired and converted to WAV digital format. Each segment might contain one or several respiratory cycles. The total duration of the segmented data was 1935s.

### 2.2. Pre-Processing

Each segment was manually trimmed from its original recording to contain one complete bout of breathing, wheezing, or coughing event. Due to the variability of recording devices, the number of channels in each segment was different. These segments were standardized to mono signals by averaging all channels. Following this, a band-pass filter was implemented, as different respiratory sounds only exhibit frequency peaks in certain regions, and signals outside the range are likely noise. For wheeze, a frequency range of spectral peaks was reported as 80–1600 Hz [19]; whereas for cough sounds, the frequency components were found to occur up to 3.0 kHz, with the majority of peaks appearing at 100–900 Hz for various cough types [15]. In this study, the band-pass filter was designed to only keep 80–3000 Hz signals.

### 2.3. Proposed Distance Function

In the choice of machine learning algorithms, it is essential to achieve both accuracy and computational simplicity to be applicable for wearable devices. Considering a sound sample of length t, $\tilde{x}=\left({\tilde{x}}_{1}\;,{\tilde{x}}_{2},\ldots {\tilde{x}}_{t}\right)$, the detection task can be generalized as:

(1) $$\underset{k}{\mathrm{argmax}\;}l_{k}\left(\tilde{x},{\tilde{c}}_{k}\right)$$

where k∈(1,2,3) are three detection classes (cough, wheeze, and breath), ${\tilde{c}}_{k}$ are the representative signals of each class, and $l_{k}$ are the respective likelihood functions. In this study, the negative distance between the sample and cluster center was used to assess the likelihood; hence, the problem can be rewritten as a minimum distance classification (MDC) problem:

(2) $$\underset{k}{\mathrm{argmin}\;}D\left(\tilde{x},{\tilde{c}}_{k}\right)$$

Note that D can be any distance metric. However, as $\tilde{x}$ is a time series with unequal length, directly measuring the distance is time consuming and computationally expensive [20,21]. Instead, a kernel-like trick was proposed to transform the segmented frames from the time series into well-established high-dimensional acoustic features, $x_{i}=\left(x_{i,1},\ldots ,x_{i,j}\right)$, where i is the ith segment of $\tilde{x}$. Various temporal and spectral features were extracted as cough and wheeze sounds have significant features in both time and frequency domains [22,23]. In the spectral domain, extracted features included mel-frequency cepstral coefficients (MFCC), spectral roll-off, spectral entropy, etc. Temporal features included amplitude and change to the energy level. In total, j=72 features were extracted in the sequence, as shown in Table 2. This feature set constructed the feature mapping from the time series to the static high-dimensional feature space.

Equation (2) is a simple mathematical model, with essential importance on the choice of distance metric D, as it needs to capture the distinct characteristics of three classes of acoustic signals in each feature while satisfying the restriction of low computational power available on wearable devices. To capture the unique characteristics of different signals in each feature, the distance function D should consider not only the absolute distance between the feature value $x_{i}$ and cluster center $c_{k}=\left(c_{k,1},\ldots ,c_{k,j}\right)$, but also the feature importance, variance, and distribution (see details in Supplementary Part A). For any feature j, such a distance metric between a sample x and an output class center $c_{k,j}$ is defined as:

(3) $$D_{j}\left(x,c_{k}\right)=\frac{\left|x_{i,j}-c_{k,j}\right|w_{j}}{\sigma_{k,j\;}\left(1+\rho \left(x_{i,j},c_{k,j}\right)\right)}\;$$

where $\sigma_{k,j\;}$is the standard deviation of jth feature of class k, and $\rho \left(x_{j},c_{k,j}\right)$ is the probability density matrix that the feature value $x_{i,j}$ belongs to class k evaluated using the histograms in training data.

Incorporating $\sigma_{k,j\;}$ into the denominator avoids the distance scaling issue where different classes and features have different variances. $\rho \left(x_{i,j},c_{k,j}\right)$ is similar to the concept of the value difference matrix in memory-based reasoning [24], defined as the probability that a sample belongs to class k when only considering feature j. As this probability density only considers a single feature at a time, this assumption seems to be similar to a naïve Bayesian classifier. However, the introduction of feature weight $w_{j}$ mitigates the impact of such an assumption: when the feature is highly correlated with other features, $w_{j}$ decreases to minimize the correlation bias. Hence, $w_{j}$ can be evaluated using the game theory approach by comparing the detection accuracy before and after the addition of feature j:

(4) $$\omega_{j}=1-\frac{\;p_{-j}\;}{p}\;$$

where *$p_{-j}$* and p are the detection accuracies with/without feature j.

### 2.4. Proposed Feature Selection Algorithms

As there were 72 features extracted, using all features on wearable devices is power consuming and unnecessary. Furthermore, the classifier is more likely to overfit the training data. For robustness and generalizability, we want the classifier to be as simple as possible, using the fewest number of features (Occam’s razor). There are various feature reduction methods available to evaluate the significance of the extracted features. In this study, we proposed a tailored feature reduction/selection method using the proposed distance function.

A better feature selection method should reflect the real discriminant power of features with respect to all output classes; meanwhile, it should consider the interactions between combined features. To achieve this, a recursive feature selection algorithm was proposed by integrating the proposed distance function in Equation (2). To reflect the real discriminant power of features with respect to all output classes and consider the interactions between combined features, the proposed method selects the feature subsets that minimize the distance from the training sample to the center of its class while maximizing distance from other classes. Let $SS_{d}$ be the collection of all feature subsets with cardinality d, containing any feature subset $S_{d}\in SS_{d}$, and $x_{i}$ be the feature vector of the ith training data segment ($x_{i}=(x_{i,1},x_{i,2},\ldots ,x_{i,72})$. The proposed algorithm selects the best feature subset $S_{d}$ from $SS_{d}$ in four steps. First, it takes one feature subset $S_{d}$ and calculates the distances $D_{k}\left(x_{i},c_{k},S_{d}\right)$ from each feature vector $x_{i}$ to output classes $c_{1},\;c_{2}$, and $c_{3}$ according to Equation (3). Secondly, it labels each feature vector $x_{i}$ with the class that has the minimum distance, denoted by $\underset{k\in 1,2,3}{\mathrm{argmin}}D\left(x_{i},c_{k},S_{d}\right)$. Thirdly, it compares the labeled classes to the ground truth for all samples and determines the accuracy of the features subset $S_{d}$. Finally, it repeats the first three steps for other candidate feature subsets $S_{d}$ in $SS_{d}$ and detects the feature subset with the highest accuracy. This process can be expressed as:

(5) $$\underset{S_{d}\in {SS}_{d}}{\max}\overset{n}{\underset{i=1}{\sum }}1\left(D\left(x_{i},c_{x_{i}},S_{d}\right)==\underset{k\in 1,2,3}{\min}D\left(x_{i},c_{k},S_{d}\right)\right)\;$$

where $c_{x_{i}}$ is the real output class of feature vector $x_{i}$ and $1\left(D\left(x_{i},c_{x_{i}},S_{d}\right)==\underset{k\in 1,2,3}{\min}D\left(x_{i},c_{k},S_{d}\right)\right)$ is an indicator function that returns 1 if two distances are equal and returns 0 otherwise.

When selecting the best feature subset $S_{d}$ containing d features, the above calculation must be repeated for $\left(\begin{array}{c} 72\\ d \end{array}\right)$ times to exhaust all feature subsets from $SS_{d}$. This could be time-consuming. To shorten this process, a recursive algorithm was proposed to start with a small number of features and expand the subsets by gradually adding more features into feature subsets. The stepwise algorithm (Algorithm 1) is as follows:
**Algorithm 1:** Recursive Feature Selection1: ***Step 1**: Start with*
m=1, *and find the best*
$S_{m}$
*using Equation (5) from all feature subsets* 2: $SS_{m}$. 3: ***Step 2**: For*
m≤d, *sort all feature subsets in*
$SS_{m}$
*by the objective function in Equation (5)*. *4: Select only the first*
$min\left(l,\left(\begin{array}{c} 72\\ m \end{array}\right)\right)$
*feature subsets based on performance, where*
l
*is the user*-5: *defined quota. In this study*, l
*is set to be 500*. 6: ***Step 3**: For every selected subset*
$S_{m}$
*from Step 2, find the absolute complement of*
$S_{m}$, 7: *represented by*
${S^{\prime }}_{m}$. *For each feature*
$f_{i}\in {S^{\prime }}_{m}$, *create a new subset*
$S_{m+1}$
*by adding*
$f_{i}$ 8: *to*
$S_{m}$; *and keep this new subset*
$S_{m+1}$
*only if its performance is better than*
$S_{m}$. 9: ***Step 4**: Insert all remaining*
$S_{m+1}$
*from step 3 to new feature set*
$SS_{m+1}$. *Increase m by 1.* 10: ***Step 5**: Repeat step 2 to 4 until*
m=d.

With the designed distance metric and the feature selection algorithm, the kernel-like minimum distance classifier (K-MDC) can be written as:

(6) $$\underset{k}{\mathrm{argmin}\;}D\left(x_{i},c_{k},S_{d}\right),\;\mathrm{where}\;\;D\left(x_{i},c_{k},S_{d}\right)\;=\sum_{j\in S_{d}}\frac{\left|x_{i,j}-c_{k,j}\right|\omega_{j}}{\sigma_{k,j\;}\left(1+\rho \left(x_{i,j},c_{k,j}\right)\right)}\;$$

In essence, K-MDC transforms the original one-dimensional acoustic time series into a high-dimensional static feature space (like the kernel trick in support vector machines). Finally, the feature space is further reduced from high dimensional to d-dimensional to optimize the classification accuracy and robustness.

### 2.5. Proposed Embedded Architecture for K-MDC Implementation

**Figure 1:**
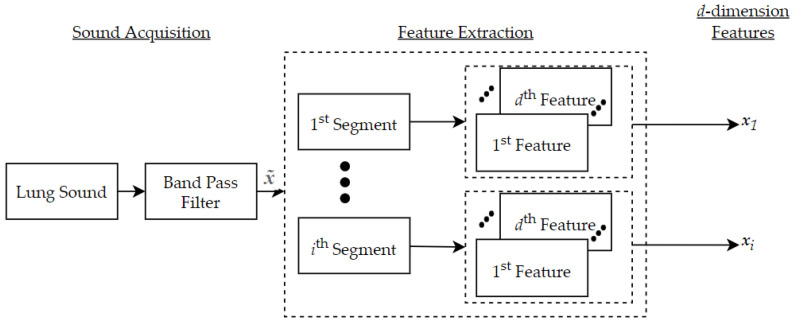
Proposed embedded architecture for the K-MDC feature extraction.

**Figure 2:**
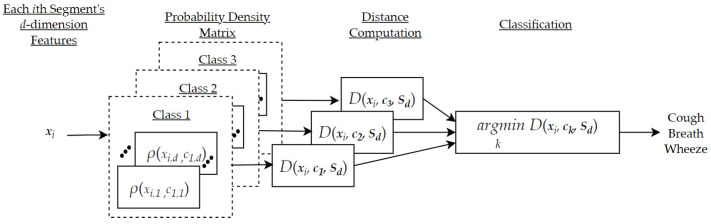
Proposed embedded architecture for the K-MDC classification of the ith segment.

After identifying a d-optimized feature space from the original one-dimensional acoustic training data samples, K-MDC can be implemented on an embedded solution using only the necessary d features extraction. Concretely, we proposed the following embedded architecture where we split the processing into two steps: (a) feature extraction, as shown in Figure 1, and (b) segment classification, as shown in Figure 2

## 3. Results and Discussion

To evaluate the proposed K-MDC, experiments were designed and conducted the study as follows. First, detection accuracy was assessed in various output classes by increasing the model complexity. Next, K-MDC was compared with the state-of-art algorithms in the mapped high-dimensional feature space. Last, the impact of the feature reduction algorithm was explored both quantitatively and qualitatively. In this section, the quantitative results were reported based on five-fold cross validation. Additionally, a demonstration of implementation in an embedded wearable based on the Renesas synergy internet-of-things (IoT) platform was evaluated.

### 3.1. Performance of the K-MDC in Various Output Classes

**Figure 3:**
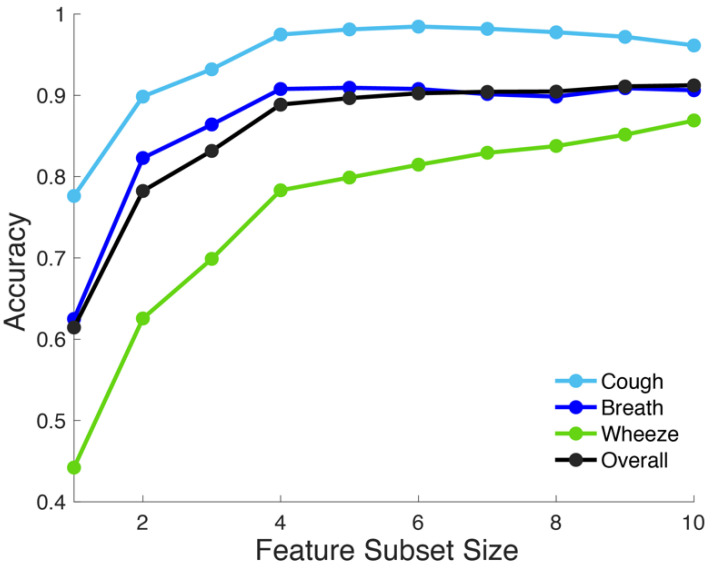
Classification accuracy of the proposed K-MDC while increasing number of features from k=1 until 10.

To evaluate the performance of K-MDC in three types of acoustic signals, prediction accuracies were recorded while increasing feature subset size of $S_{d}$ from d=1 to 10. Feature subsets were gradually expanded by the proposed recursive feature selection algorithm in Section 2.4. The detection accuracies for each output category as well as overall performance were plotted in Figure 3. K-MDC was able to achieve overall detection accuracy of more than 90% and it delivered the best detection performance for cough data (≥95% when d≥4). The detection accuracies of all classes increased more drastically when d≤4, and tended to taper as the number of features further increased. When d=10, the overall detection accuracy was 91.23%.

### 3.2. Comparison with State-of-Art Algorithms

**Figure 4:**
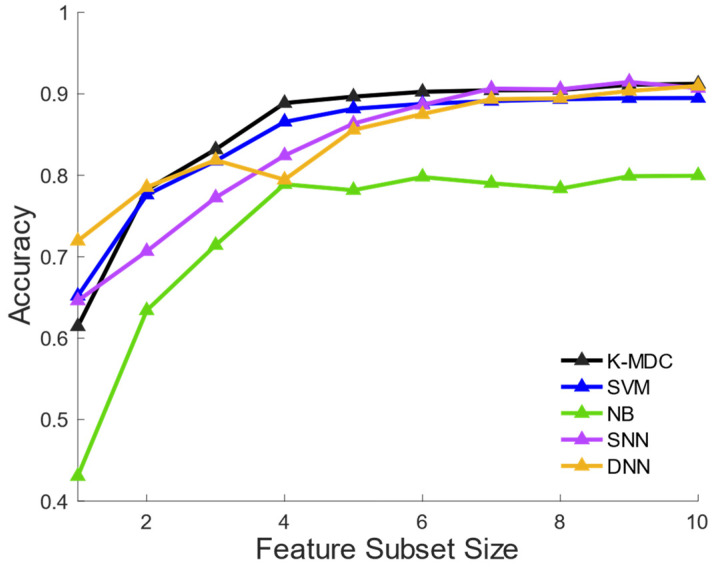
Performance comparisons between K-MDC, SVM, SNN, DNN, and NB while increasing the number of features.

Next, the proposed K-MDC was compared with state-of-art machine learning algorithms which include naïve Bayes (NB), support vector machine (SVM), and artificial neural network (ANN), which are widely used classifiers in acoustic signal processing. To study the effects of deep learning in this detection problem, two variants of ANN models were implemented: a shallow neural network (SNN) with 1 hidden layer of size 10 and a deep neural network (DNN) with three hidden layers of sizes (128, 64, and 32). MATLAB Statistics and Machine Learning Toolbox and MATLAB Neural Network Pattern Recognition Toolbox were used for implementing DN, SVM, and SNN, and Python Pytorch [25] was used for implementing DNN. The comparison of K-MDC with baselines was recorded at various feature dimensionalities in Figure 4. It shows that the proposed K-MDC method achieved superior performance compared with the implemented machine-learning classifiers in most cases, implying its capability of distilling predictive information from limited feature dimensionality. When comparing SNN with DNN models, both models achieved similar prediction accuracy with a larger number of input features. When given a smaller number of input features, DNN was more capable of distilling information and produced better prediction than SNN when d≤3. However, such complicated architecture was less stable and was outperformed by SNN when 4 ≤d≤9.

As the requirement of smaller features implies a shorter computation time and power for feature extraction and classification (hence faster detection speed and working hours) on wearable devices, it is interesting to investigate the number of features required to achieve certain levels of prediction accuracy for each machine-learning classifier. Moreover, simpler models also lead to more robustness and better generalizability, according to Occam’s razor. The minimum required feature dimensionality to achieve prediction accuracy of 80%, 85%, and 90% for each classifier, respectively, was measured. In addition to the abovementioned classifiers, this test was extended to K-nearest neighbors (KNN) using MATLAB Statistics and Machine Learning Toolbox. The optimal number of K in KNN was found to be K = 18, and the results of all classifiers were recorded in Table 3. As can be seen, K-MDC reached various accuracy thresholds with the smallest number of required features. This implies the best distillation and utilization of feature information in K-MDC as it takes variance, density distribution, and mutual information of each class and each feature into consideration. The sensitivity analysis of each feature in the K-MDC model was conducted in Supplementary Part B using the game theory approach.

### 3.3. Performance of Feature Selection

Performance of the proposed feature selection algorithm was both quantitatively and qualitatively evaluated. Table 4 lists the average number of features required for each machine-learning algorithm to converge to its optimal accuracy, where the convergence was identified when the accuracy was less than 3% away from the highest accuracy. Note that the highest testing accuracy may not be attained with the highest dimensionality because the higher dimensionality leads to more model complexity and overfitting. To exemplify this, we calculated the accuracy difference between the model constructed with the proposed feature selection method and the model constructed using all features. As shown in Table 4, these classifiers converge to their optimal performance with fewer than 8 features out of 72 features with the proposed feature selection method. The best performance improvement (in the case of K-MDC) was up to 13.24% when compared with the performance using randomly selected features with the same dimensionality. However, the ANN models (both SNN and DNN) with feature selection did not perform better than without feature selection, even though feature selection reduced total computation effort. This is due to the nature of the ANN architecture with early stopping, and such a finding is in accordance with other studies using different datasets [26]. Therefore, great caution must be taken when performing dimension reduction techniques with neural networks to consider the trade-off between computation time and detection accuracy. Computation complexity analysis of the proposed model and other baselines was discussed in Supplementary Part C.

Compared with other feature selection methods, for example, principal component analysis (PCA), the proposed method also performed better. PCA selected spectral roll-off ($f_{40})$ and energy variation ($f_{54})$ as the best two-feature combinations, contributing to a total of 99.57% of its explained variance. When using these two features for classification, it yielded an overall detection accuracy of 72.28%, which was inferior to the selected two-feature combination ($f_{2}$ and $f_{53}$) in the proposed algorithm with an accuracy of 85.20%.

**Figure 5:**
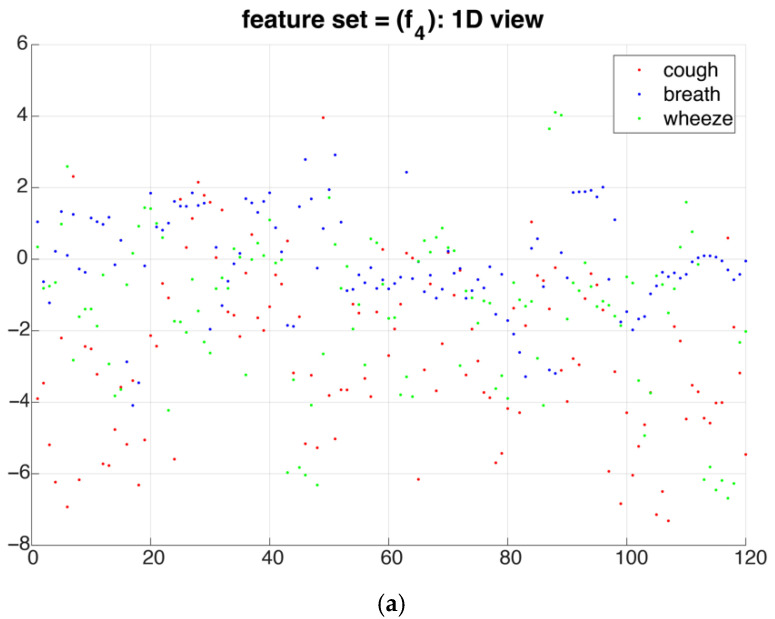

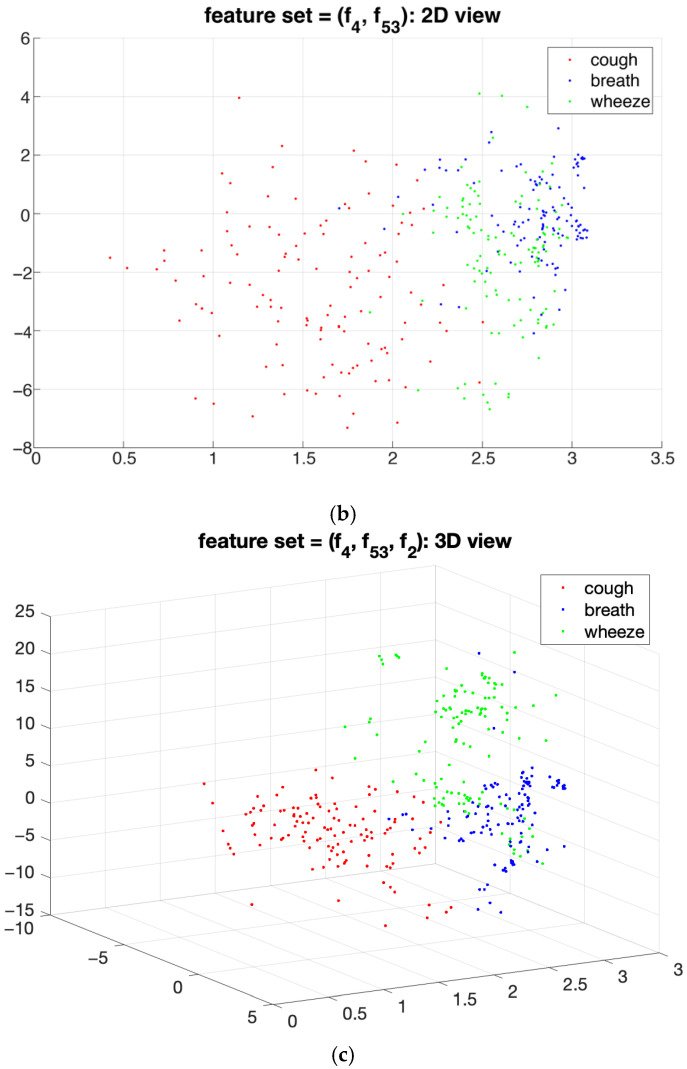
Distribution of cough, breath, and wheeze data in (**a**) 1-dimensional feature space where the x-axis is the sample index, (**b**) 2-dimensional feature space, and (**c**) 3-dimensional feature space. The feature space is constructed by recursively adding features into feature subsets according to Algorithm 1. Each dot represents an acoustic segment of cough (red), breath (blue), or wheeze (green) sound.

To qualitatively visualize the superiority of the proposed method, Figure 5 illustrates the clustering of samples by recursively expanding the feature space from one dimensional to three dimensional. Training samples belonging to different classes were initially hard to distinguish when only one feature was chosen from the feature subset. In subsequent steps, the proposed algorithm selected one feature each time to optimize the objective function and the separation between different classes became increasingly distinct.

### 3.4. Implementation of the Proposed Algorithm in Embedded Systems

**Figure 6:**
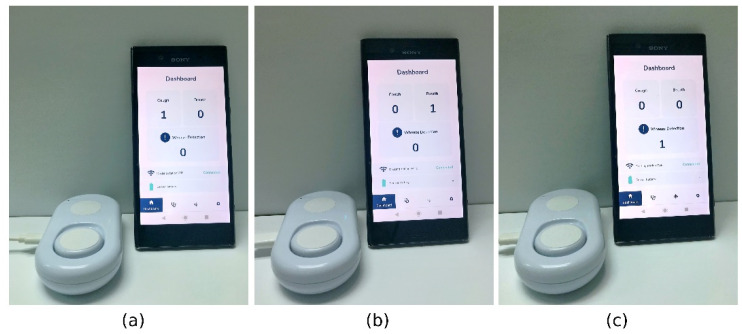
K-MDC classification of (**a**) cough, (**b**) breath, and (**c**) wheeze running in an IoT environment using the Renesas.

Finally, we implemented the K-MDC using the proposed architecture described in Section 2.5 onto an embedded system based on Koh et al. [27] running on the Renesas S5D9 120 MHz Arm^®^ Cortex^®^-M4 CPU in an IoT environment. The classification results from K-MDC are published on an IOT cloud, and the results are extracted through a smartphone connected to the same IOT cloud. Specifically, in Figure 6, we show the results for the classification of (a) cough, (b) breath, and (c) wheeze. In all cases, the classified sounds are preloaded in the embedded system as the main purpose is to determine the feasibility of K-MDC in a wearable device.

## 4. Conclusions

This study proposed a kernel-like detection algorithm for classifying acoustic time series into cough, wheezing, and breathing. It has a novel distance measure that considers each feature’s unique properties, including the class variance, probability distribution, and feature importance. Results showed that the proposed method achieved better detection accuracy than existing algorithms, and the proposed feature reduction method effectively reduced the feature dimensions to 4 from 72 and, at the same time, improved the overall classification by 13.24%. In wearable device applications, feature dimensionality is of concern as a smaller number of features requires shorter computation time for feature extraction and classification (faster detection speed) and hence less consumption of battery power (longer working hours). From our findings, the proposed method potentially tackles the current challenges of limited processing power by minimizing the feature extraction efforts and detection accuracy in health-monitoring wearable devices by capturing the unique distribution characteristics in different features and sound classes. This is also exemplified in this work through the implementation of the proposed algorithm in an embedded platform based on the IOT chipset from the Renesas synergy series. Furthermore, the findings in this study may be applied to other health-monitoring applications, including the quantification and severity analysis of clinical symptoms.

## Figures and Tables

**Table 1:** Constitution of input data sources.

Data Source	Percentage (Total Length)
Hospitalized patients	44.9%
Recruited subjects	17.3%
Open dataset	37.8%

**Table 2:** Summary of extracted features.

$\mathrm{Features}\;f_{k}$	Type	Number of Features
$f_{1}\sim f_{13}$	MFCC coefficients	13
$f_{14}\sim f_{26}$	First derivatives of MFCC coefficients	13
$f_{27}\sim f_{39}$	Second derivatives of MFCC coefficients	13
$f_{40}$	Spectral roll-off	1
$f_{41}\sim f_{49}$	Power spectral density	9
$f_{50}$	Spectral entropy	1
$f_{51}$	Amplitude	1
$f_{52}$	Spectral flatness (measured by variance)	1
$f_{53}\sim f_{54}$	Energy variations	2
$f_{55}\sim f_{72}$	Dominance of frequency bands	18

**Table 3:** Minimum required number of features to achieve specific detection accuracies. ‘-’ indicates that the classifier could not achieve the required accuracy threshold using our dataset.

Thresholds	K-MDC	SVM	NB	SNN	DNN	KNN
Accuracy ≥ 80%	2	2	4	4	3	3
Accuracy ≥ 85%	2	3	-	5	5	3
Accuracy ≥ 90%	4	4	-	5	9	4

**Table 4:** Performance difference with or without feature selection methods and required number of features for the detection accuracy to converge, where the convergence is defined as less than 3% from the optimal accuracy.

Classifiers	Performance Difference	Required Number of Features
K-MDC	13.24%	4
SVM	0.82%	7
NB	12.58%	4
SNN	−6.50%	5
DNN	−1.08%	6
KNN	5.30%	7

## Data Availability

Not applicable.

## Supplementary Materials

The following supporting information can be downloaded at: https://www.mdpi.com/article/10.3390/s22062167/s1, Figure S1: Variances of features by class. For easy comparison, the variances of breath are scaled to 1 for all features; Figure S2. The distribution patterns of features by classes. Bar shades are the relative frequencies for each class and curves are the fitted probability density distributions. Figure S3. Bimodal and multi-modal distributions of features in three classes. Bars are the relative frequencies for each class and curves are the fitted probability density distributions. Figure S4. Sensitivity analysis of features in each outcome and overall accuracy. Performance change is measured by the improvement (if any) when the current feature is included in the model. Table S1: Computation complexity of machine learning classifiers using Big O notation. n is the size of training data, k is the number of selected features, and K is the number of nearest neighbors in KNN. ‘-’ implies that the computation complexity is not applicable or cannot be directly expressed [28,29].
