# Supplementary material for: Distance-Based Detection of Cough, Wheeze, and Breath Sounds on Wearable Devices
Source: Sensors (Basel). 2022 Mar 10;22(6):2167. doi: 10.3390/s22062167 (PMC8950004; doi:10.3390/s22062167)
Supplement: Supplementary file 1 [file sensors-22-02167-s001.zip › sensors-1552426-supplementary.pdf]

sensors

Distance-Based Detection of Cough, Wheeze, and Breath Sounds on Wearable Devices

Bing Xue [1], Wen Shi [2], Sanjay H. Chotirmall [3], Vivian Ci Ai Koh [4], Yi Yang Ang [4], Rex Xiao Tan [4] and Wee Ser [4,*]

[1] Department of Electrical and Systems Engineering, Washington University in St. Louis, St. Louis, MO 63130, USA; xuebing@wustl.edu
[2] Harvard Medical School, Harvard University, Cambridge, MA 02115, USA; wshi3@mgh.harvard.edu
[3] Lee Kong Chian School of Medicine, Nanyang Technological University, Singapore 637551, Singapore; schotirmall@ntu.edu.sg
[4] Aevice Health Pte. Ltd., Singapore 637551, Singapore; vivian@aevice.com (V.C.A.K.); yiyang@aevice.com (Y.Y.A.); rex@aevice.com (R.X.T.)
* Correspondence: wser@spacemedtech.com or serw01@gmail.com

Part A:

Modelling the uniqueness of each class in each feature is the key challenge in multi-class detection in acoustic biomedical signals. To fully explain this issue and justify our approach, here we divide it into two sections to explain the motivations of including variance and distribution separately.

Inclusion of variance into the model

The incorporation of variance of features into the model (instead of using standard normalization) has two key reasons: 1) the feature variances in different classes are very different, implying a high inter-class variance ratio; and 2) each feature has its unique inter-class variance ratios. To validate this, we first calculated the variance of each feature in each class. Next, we calculated and plotted (see Figures S1 in this response letter) the inter-class variance ratios $\frac{\sigma_{j,k=wheeze}}{\sigma_{j,k=breath}}$ (in red) and $\frac{\sigma_{j,k=cough}}{\sigma_{j,k=breath}}$ (in blue) for all features ($j = 1, \dots, 72$). As we can see, for almost all features, the variances in 3 classes are quite different. Such inter-class variance ratio can even go as high as >1e6 (see Figures S2 and S3 for the distributions of some features). Therefore, it is necessary to incorporate the variance information at the class level. Moreover, since the inter-class variance ratio is not constant across different features –the ratios in some features are more drastic than others, implying the necessity to incorporate the variance at the feature level too.

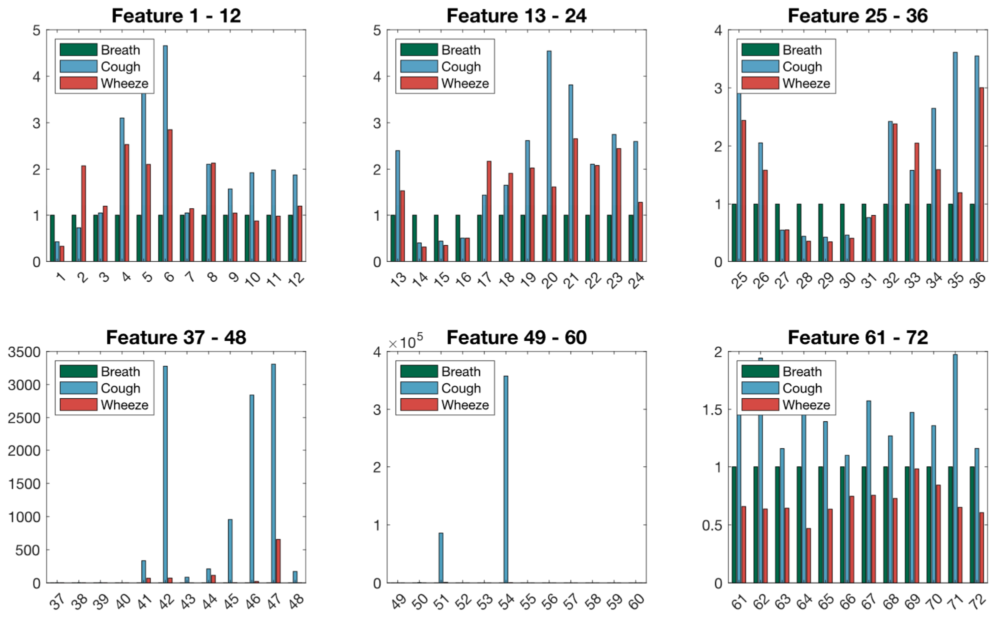

Figure S1. Variances of features by class. For easy comparison, the variances of breath are scaled to 1 for all features.

Inclusion of distribution into the model

The motivations of including distribution into the models are two-fold: a) the distribution types are different in different features/classes, and b) using mean/variance cannot capture the multimodality of class centroids. To see a), we randomly picked a few features and demonstrated the distribution differences between 3 classes in Figure S2. On the left subplot, cough and breath sounds are skewed to the right but wheeze is highly skewed to the left; on the right subplot, cough sounds are flat, but breath and wheeze are squeezed to the right tails. As these distribution patterns vary by output classes as well as features, the detection algorithm should characterize the uniqueness of each class in each feature.

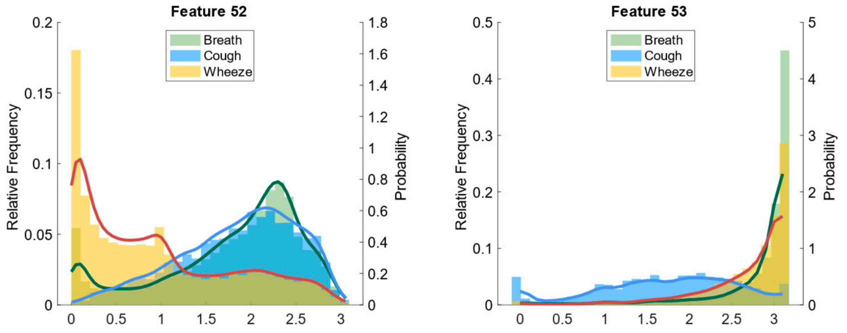

Figure S2. The distribution patterns of features by classes. Bar shades are the relative frequencies for each class and curves are the fitted probability density distributions.

When the feature values follow a simple distribution (for example, Gaussian), then incorporating mean and variance into the model might be sufficient to calculate the distances to the class centroids. However, this is not true in our detection problem. In fact, the feature values are distributed in bimodal or multimodal fashion (see Fig. 3). On the left subplot, we showed the multimodal patterns in breath sounds; in the middle subplot, we showed the bimodal distribution of wheeze, and in the right subplot, we showed the bimodal distribution of both cough and non-cough sounds. In view of the multimodality, we have adopted the probabilistic measure into our model by learning the empirical distribution patterns from historical data. By doing so, the proposed model has a better representation of the relative between a sample and the class centroids.

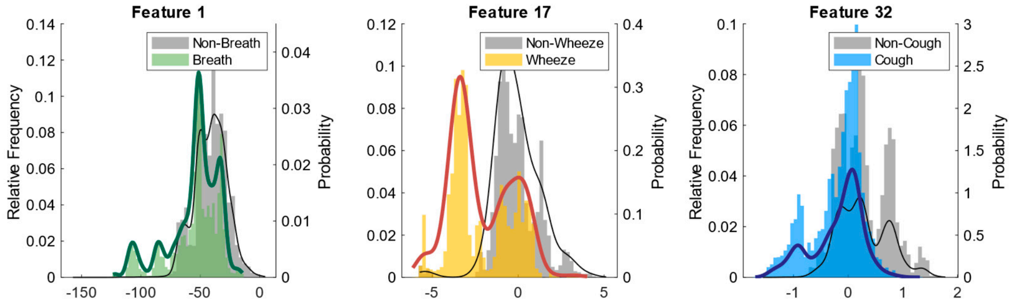

Figure S3. Bimodal and multi-modal distributions of features in three classes. Bars are the relative frequencies for each class and curves are the fitted probability density distributions.

Part B:

Here we investigate the feature sensitivity in 3 outcomes and the overall prediction (see Figure S4), using a game theory approach, designed as follows:

For each feature:

1. Remove the feature from current dataset, and build a K-MDC model. Record the average model performance in 3 outcomes and the overall detection accuracy.
2. Build another K-MDC model by forcing the model to take this feature. Record the average model performance in 3 outcomes and the overall detection accuracy.
3. Calculate the improvement of model performance (if any) when this feature is forced to be included.

As we can see, the wheeze prediction appear to be more sensitive to adding/removing certain features, while other outcomes are more robust.

However, as K-MDC learns to minimize the mutual information when building models from the features, adding/removing a single feature has little impact to the overall prediction performance, where the largest improvement in detection accuracy is less than 2.5%.

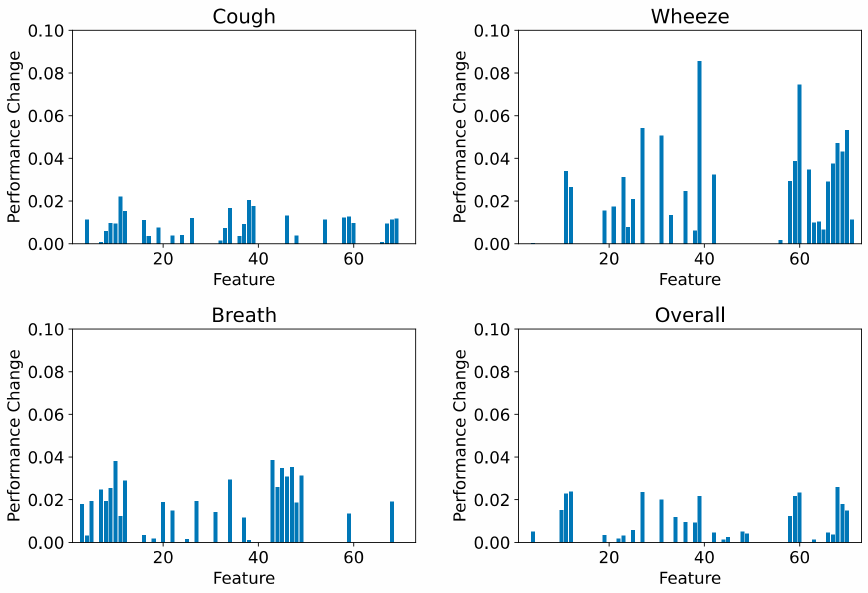

Figure S4. Sensitivity analysis of features in each outcome and overall accuracy. Performance change is measured by the improvement (if any) when the current feature is included in the model.

Note that the sensitivity analysis is not covered in the main context, mainly because of the heterogeneity of sound signals. For instance, the cough sounds can come from various airway diseases with various spectral patterns (Debreczeni et al. 1987), hence the sensitivity to these spectral features will be largely dependent on the prevalence of diseases in the collected sounds. A systematic and conclusive sensitivity analysis would require large acoustic data from various airway diseases, which is not feasible in the current study.

Part C

We have performed the computational complexity analysis. From the Equation 5 and Equation 6, we can see that the computation complexity of training is proportional to the amount of training data and the square of feature size and the computation complexity of prediction is proportional to number of features. The computation complexity of various models is summarized below in Table S1. Note that due to the design and implementation, the computation complexity of SVM models can be quite different. Here we report the results by Fleizach and Fukushima (1998) and Abdiansah and Wardoyo (2015). It is obvious that computation complexity of prediction stage is more critical because training stage can always be conducted offline but classification has to be repeated continuously on wearable devices. Therefore, our proposed K-MDC ($O(k)$) is computationally more efficient than SVM and KNN, and equivalent to NB. Note that the

computation complexity of ANN is largely dependent on the number of hidden layers and the architecture, but is generally more computationally expensive than other classifiers.

Table S1. Computation complexity of machine learning classifiers using Big O notation. n is the size of training data, k is the number of selected features, and K is the number of nearest neighbors in KNN. '-' implies that the computation complexity is not applicable or cannot be directly expressed.

Classifier	Training	Classification
**K-MDC**	$O(nk^2)$	$O(k)$
**ANN**	-	-
**NB**	$O(nk)$ [29]	$O(k)$
**SVM**	$O(kn^2)$ to $O(kn^3)$ [28]	$O(k)$ to $O(kn)$
**KNN**	-	$O(kK)$
